# Call for Papers, Issue 3/2023: Home Office: Working from a Private Place

**DOI:** 10.1007/s12599-021-00699-8

**Published:** 2021-06-08

**Authors:** Mark Ackerman, Alexander Maedche, Claudia Mueller, Gerhard Schwabe, Volker Wulf

**Affiliations:** 1 University of Michigan, Ann Arbor, MI USA; 2 Karlsruhe Institute of Technology (KIT), Karlsruhe, Germany; 3 University of Siegen, Siegen, Germany; 4 University of Zurich, Zurich, Switzerland

## Special Issue

The availability of digital technologies offers the opportunity to reshape work and life. In particular, the traditional spatial and temporal restrictions for workspaces are increasingly being reduced. Even beyond COVID-19, many office employees will continue to work, at least partly, from home. Although the degree of virtualization will continue to increase, a combination of physical and virtual presence can be expected. Hybrid forms of working will become part of our future private and professional life.

Working from home goes hand in hand with great potential, but at the same time also with risks. Recent experiences with home schooling and home office have triggered intensive discussions on potential positive and negative outcomes of co-locating work with private and schooling activities. There is a need to design digital technologies appropriately to support individuals, groups and organizations in ways that increase productivity and wellbeing. Thus, pursuing a socio-technical paradigm for understanding and designing for the home office is essential. The fields of Information Systems (IS), Computer-Supported Cooperative Work (CSCW), and Human–Computer Interaction (HCI) have a long tradition in designing from a socio-technical perspective. Building on this tradition, we believe that existing work practices in the home office need to be analyzed and understood more intensively. Digital technologies must be designed and tailored to fit into the complexities of the home office. New descriptive and prescriptive knowledge must be provided to fully leverage the potential of working from home.

This special issue welcomes a diversity of submissions and is hence open for empirical, design-oriented, and conceptual research focusing on working from home. Manuscripts may employ qualitative, quantitative, engineering, mixed methods, or innovative research designs. They may address the individual, group or organizational level.

Thus, topics may include, but are not limited to, the following areas:

- Empirical studies of work practices and social practices in home offices
- Innovative designs of digital technologies in support of home office work, e.g., NeuroIS (e.g., eye-tracking-based or physio-adaptive IS), conversational agents or AR-/VR-based IS
- Methods and techniques for implementing and operating home offices
- New conceptualizations and theories of working from home
- Individual-, group-, organization-, and multi-level studies of home office working antecedents and outcomes from a social, psychological, and performance perspective
- Appropriation of digital technologies for working from home
- Studies on innovative hybrid home office designs and corresponding office designs
- Leading and managing working from home
- Gender studies on working from home
- Studies on control and surveillance and its avoidance in home offices
- Studies on hybrid service provision from home

All submissions should cover digital aspects of working from home and open the "black box" of information technology (i.e., shed light on specific aspects of the applied IT artifacts).

## Submission Guidelines

Please submit your paper by 1 December 2021, via BISE’s online submission system (https://www.editorialmanager.com/buis/). Please observe the instructions on manuscript formatting and length. Submission guidelines and general author guidelines are available at http://www.bise-journal.com/author_guidelines.

Each submission will be reviewed anonymously (double-blind process) by at least two referees with respect to its relevance, originality, and research quality. In addition to the editors of the special issue, distinguished international scholars will be involved in the review process as associate editors.

## Schedule

- Paper submission due: 1 December 2021
- First notification of authors: 1 February 2022
- Completion of first revision: 1 May 2022
- Second notification of authors: 16 July 2022
- Completion of second revision: 1 September 2022
- Online publication: ASAP
- Anticipated print publication: Issue 3/2023

